# Porosity Measurement of Low Permeable Materials Using Gas Expansion Induced Water Intrusion Porosimetry (GEIWIP)

**DOI:** 10.1038/s41598-019-53441-6

**Published:** 2019-11-26

**Authors:** Miad Jarrahi, Douglas W. Ruth, Mohamed T. Bassuoni, Hartmut M. Holländer

**Affiliations:** 1 Department of Civil Engineering, University of Manitoba, Winnipeg, Manitoba Canada; 2 Department of Mechanical Engineering, University of Manitoba, Winnipeg, Canada

**Keywords:** Solid Earth sciences, Core processes, Engineering, Civil engineering, Characterization and analytical techniques

## Abstract

Porosity measurement is a key factor to identify the hydraulic performance of low permeable porous materials (e.g. rock or concrete). Porosimetry tests such as Mercury Intrusion Porosimetry (MIP), Nuclear Magnetic Resonance (NMR), or Gas Expansion (GE) are cost-prohibitive, use hazardous materials, or are incapable of accessing all inter-connected pores. An alternative Gas Expansion Induced Water Intrusion Porosimetry (GEIWIP) method was developed to measure the porosity of a low permeable porous medium using a gas/water intrusion apparatus. This method overcomes the previously mentioned porosimetry drawbacks by using distilled de-aired water (DDW) as a hazard-free liquid which is a wetting fluid to intrude the porous structure and fill the pores. As the DDW has the tendency to fill all inter-connected pores, no back-up pressure is required. This method has lower cost and needs less preparation time comparing to MIP test. Additionally, the GEIWIP set-up, the gas/water intrusion apparatus, can be moved to the field site and provide mobile measurement feasibility. The reliability of the test results was obtained by a repetitive testing process. The porosity of concrete samples with different mixtures was obtained and compared to those of MIP and NMR tests.

## Introduction

The characterization of porous materials was carried out through many experimental techniques that measure the porosity of the solid matrix. Porosity measurement is recognized as an important evaluation of geophysical materials such as shale rock or structural materials such as concrete.

In concrete, porosity data contribute to our understanding of the nature of the concrete mixture and impacts its strength, resistivity, applicability, and service life. The pore size distribution (PSD) in concrete is associated with the transport processes of moisture and aggressive ions, which can cause alkali-silica expansion, sulfate attack, corrosion of embedded reinforcement, etc.^1^. The physical significance of PSD in concrete encompasses the “voids”, as coarse pores, the “capillary pores”, as the middle size pores, and the “gel pores”, as the fine pores^1^.

The complexity and diversity of porous materials has led to lack of general agreement on the advantages and disadvantages of the various procedures described in the literature^2^. According to IUPAC technical report^2^, the recommended methods available for porosity measurementfor consolidated porous materials are stereology, radiation scattering, pycnometry, adsorption from the gas phase, intrusion, suction, maximum bubble pressure, fluid flow, immersion or adsorption calorimetry, thermoporometry, size exclusion chromatography, Xenon NMR and ultrasonic methods^2^. A common example of intrusion method to analyse consolidated low permeable media is the Mercury Intrusion Porosimetry (MIP)^3,4^. The measurement is based on the Washburn model^5^ and evaluates the diameter of cylindrical pores filled at each intrusion pressure. However, the Washburn model is only valid for porous systems made up of cylindrical pores with specific diameters, all connected to the outer surface directly through pores with a larger diameter. Nevertheless, not every porous material conforms to this type of pore network. Concrete, as an example, is a hydrated cement-based material that is shown to have two main incompatibilities with the Washburn model: (i) pores were reported to have a high degree of convolution^6^, and considerably fractal character^7^, that can hardly be assumed to have a cylindrical shape; (ii) the interior pores were accessible to mercury indirectly through percolated chains of finer pores known as “ink-bottle” connected pores^8^. Windslow and Diamond^9^ indicated that the saturating process of cement pastes during MIP was independent of the pore sizes, thus leading to inaccurate measurements of pore size distributions using MIP.

The comparison of MIP with carbon dioxide and nitrogen low pressure gas adsorption (LPGA), and advanced Small Angle X-ray Scattering (SAXS) analytical techniques were carried out for the surface area and porosity of bituminous coals^10^. They reported that nitrogen adsorption underestimated the surface area and porosity of the samples comparing to MIP and SAXS. The SAXS provided larger porosity comparing to other techniques due to probing a wider range of pores including the closed pores^10^.

In addition, in MIP test for porous samples including dead-end pore spaces, channels, and micro-cracks in their pore structure, such as concrete, mercury may shortcut through the channels and get intruded at relatively lower pressures^8^. Moreover, according to Washburn model limitations, at each operating pressure, there might be larger pores that cannot be captured by the MIP test, as these pores are surrounded by finer pores. The equilibrium geometry of liquid-solid interfaces of mercury, a non-wetting fluid, and grain walls leads to a convex meniscus and an opposing capillary action, preventing mercury penetration into the pores^11^. This raises two main drawbacks of using mercury for porosimetry. First, due to the high surface tension of mercury, high level of back-up pressure is required to force the mercury into the void spaces. Second, only pores that are connected to the material outer surface through larger pore may be penetrated. This encourages the use of a wetting fluid in porosimetry, instead of mercury or gas, to remove the need for back-up pressure and yet to increase the capability to fill smaller pores that are inter-connected with finer poles to the surface.

In gravimetric method, water is used to determine the pores volume by measuring the volume of water that saturates the pores in vacuum condition^4^. In this method, the evacuation is done after the pores are flushed with carbon dioxide. Then, water is absorpted to the pores due to capillary action^4^.

The porosity measurement of organic-rich mudrocks were carried out with RockEval pyrolysis, subcritical gas-adsorption analysis with nitrogen, and X-ray diffraction^12^. The liquid saturation and immersion method was used to measure the total porosity of high organic matter shale rock. The water immersion porosimetry (WIP)^13^ technique was used to measure total porosity of shale samples from an Eastern Europe Silurian gas shale play and the Haynesville Shale from East Texas, USA. The results from WIP test were compared with other standard methods including the method developed by Gas Research Institute (GRI), and mercury intrusion porosimetry. In their study, the advantages, drawbacks, experimental errors, and reproducibility of WIP are presented^13^. The samples were being saturated with deionized (DI) water under constant pressure of 13.7 MPa (2000 psi) for 24 h. However, saturating those clay rich lithologies with DI water may induce swelling and disturb the bulk density measurements. Therefore, they applied a swelling test to confirm the validation of the method. In the swelling test, the average volume change after saturating process was 0.92% (±2.29%). Therefore, the effect of swelling of rock during saturating process with DI water was less than the uncertainty of the measured porosity values, showing minor error in porosimetry. However, the tests’ long time period was a disadvantage of this experiment.

Other methods are recommended^2^ for other types of porous materials as following. The pore structure features of enhanced porosity concretes was studied using stereological techniques based on on area and line fractions^14^. The helium pycnometry was used to characterize biochar porosity and the result was compared with those measured by nitrogen gas sorption and mercury porosimetry^15^. It was shown that macropores of the biochar pore structure was not detected by gas sorption analysis. The porosity of aerobic granules with different diameters were assessed using size-exclusion chromatography^16^. It was shown that the extracellular polymeric substances of the granules might clog the pores and might be responsible for underestimation of porosity^16^. The porosity of gas-bearing sandstones measured by nuclear magnetic resonance (NMR) logs was underestimated due to the effect of the lower hydrogen index of natural gas in these rocks^17^. An ultrasonic method was used in an experimental reflected waves to measure the porosity of air-saturated porous materials^18^. This study stated that porosity is sensitive to wave reflection, especially when the incident angle is less than its critical value, at which the reflection coefficient disappears^18^. Therefore, porosity as the value of the ratio of pores volume with respect to the total or bulk volume of solid depends on the method used to determine those volumes. The methods using a fluid have only access to open pores leading to apparent porosity, while methods using a radiation only detect the closed pores leading to closed porosity, or the methods with access to all pores (*e*.*g*. SAXS) lead to total porosity^2^.

One of the most accurate and rapid techniques for the determination of porosity of rocks is the method of Gas Expansion (GE)^19^. This method was used in many experimental studies such as Ernest^19^, Luffel, *et al*.^20^, and Karastathis^21^. In this method, the gas at an initial pressure was allowed to expand and fill the pores within a short time period (i.e. 5–15 min). The pore volume was obtained by measuring the corresponding pressure drop from an initial value to the final state. The governing relationship between the pressure drop and the expanded volume was derived based on Boyle’s law^22^. One drawback of the GE method is the lack of a record of change in pressure versus time so that other parameters such as permeability cannot be measured. In addition, Boyle’s law is only valid when the expansion process is isothermal, where the system is in thermodynamic equilibrium. Considering that the gas penetration is a fast occurring process, it may be unlikely to maintain the equilibrium status during the gas expansion test, thus, may deviate from thermodynamic equilibrium.

Depending on the application of the porous solid, the preferred method can be selected for porosity measurement based on its accuracy, feasibility, experimental cost, and the fluid used in that experiment. The methods using water intrusion are recommended for porosity determination of concrete structures that are in contact with water. This study is an effort to identify the porosity of concrete samples with intrusion method using a wetting fluid under gas pressure to ensure the saturation within an arbitrary time frame and that is called as Gas Expansion Induced Water Intrusion Porosimetry (GEIWIP). The time to saturate the samples was controlled by the applied initial pressure within a gas/water accumulator. This method uses the principals of intrusion^2^ and gas expansion method^19^, despite the fact that instead of gas intruding the pores, water is used to fill the pores. However, unlike the GE test, the process of saturation did not require to be isothermal. Most methods use small samples where multiple samples must be analyzed to get statistically representative. However, in this method the cylindrical samples can be as large as 50 mm in diameter as a representative of the target area, which is better than those tests with smaller limit of sample size.

In this experiment, argon gas and distilled de-aired water (DDW) were stored in an accumulator under an initial pressure. The accumulator was connected to a vessel containing the porous sample. Once DDW intruded into the pores of the sample, the gas within the accumulator got more volume to expand, thus the pressure dropped from its initial value. The change in pressure was used to calculate the saturated pores’ volume, thus the porosity. The GEIWIP method was carried out on four concrete samples with different mixtures and porosities ranging from 10 to 20%. As DDW was a wetting fluid in concrete, it had a tendency to fill the larger pores behind the finer pores, yielding larger porosity measurements comparing to MIP measurements. The validity of the test results was assessed by comparing them with modified GE, MIP, and NMR performed on the same concrete samples. A good agreement was seen with modified GE and MIP. The better agreement was obtained with NMR results.

## Methodology

### Sample characterization

Four concrete samples were prepared according to mixtures described in Table 1. Results for resistance to different de-icing salt exposures on the same concrete were published by^23^. The main binder (cement paste) was general use (GU) Portland cement, Portland limestone cement (PLC), and fly ash (Class F). A small proportion of carboniferous aggregate was used with natural gravel with a maximum size of 9.5 mm. A well-graded river sand was used as the fine aggregate in the concrete structure. More details can be found in^23^.

**Table 1:** Proportions of mixtures per cubic meter of concrete (according to^23^).

Sample ID.	Cement (kg/m^3^)	Fly Ash (kg/m^3^)	Nanosilica (kg/m^3^)	Water (kg/m^3^)	Coarse Aggregate (kg/m^3^)	Fine Aggregate (kg/m^3^)	28 day Compressive Strength (MPa)
GUF20	320	80	—	160	1077	580	38 (0.7)
GUF30	280	120	—	160	1068	575	35 (1.1)
PLCF30	280	120	—	160	1068	575	40 (0.8)
PLCF30S	256	120	48	136	1063	573	47 (0.3)

The values between parentheses in the last column are the standard errors.

**Figure 1:**
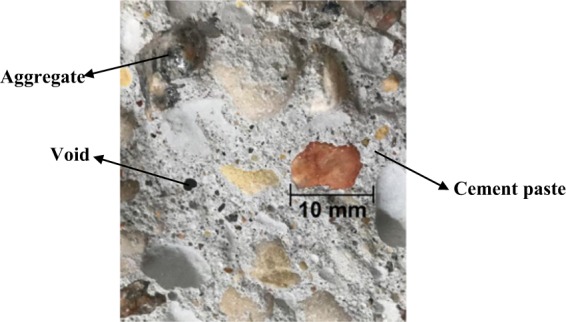
Dried concrete sample consisting of cement paste, voids, and aggregates.

In the name of the sample ID, GU or PLC referred to the type of binder, F20 and F30 expressed 20 and 30% fly ash, respectively, and S referred to nanosilica. The material mixing procedures, casting and curing conditions were described in^23^. Figure 1 shows one dried concrete sample body containing the voids, hardened cement paste, and aggregates.

### Gas/water intrusion apparatus

**Figure 2:**
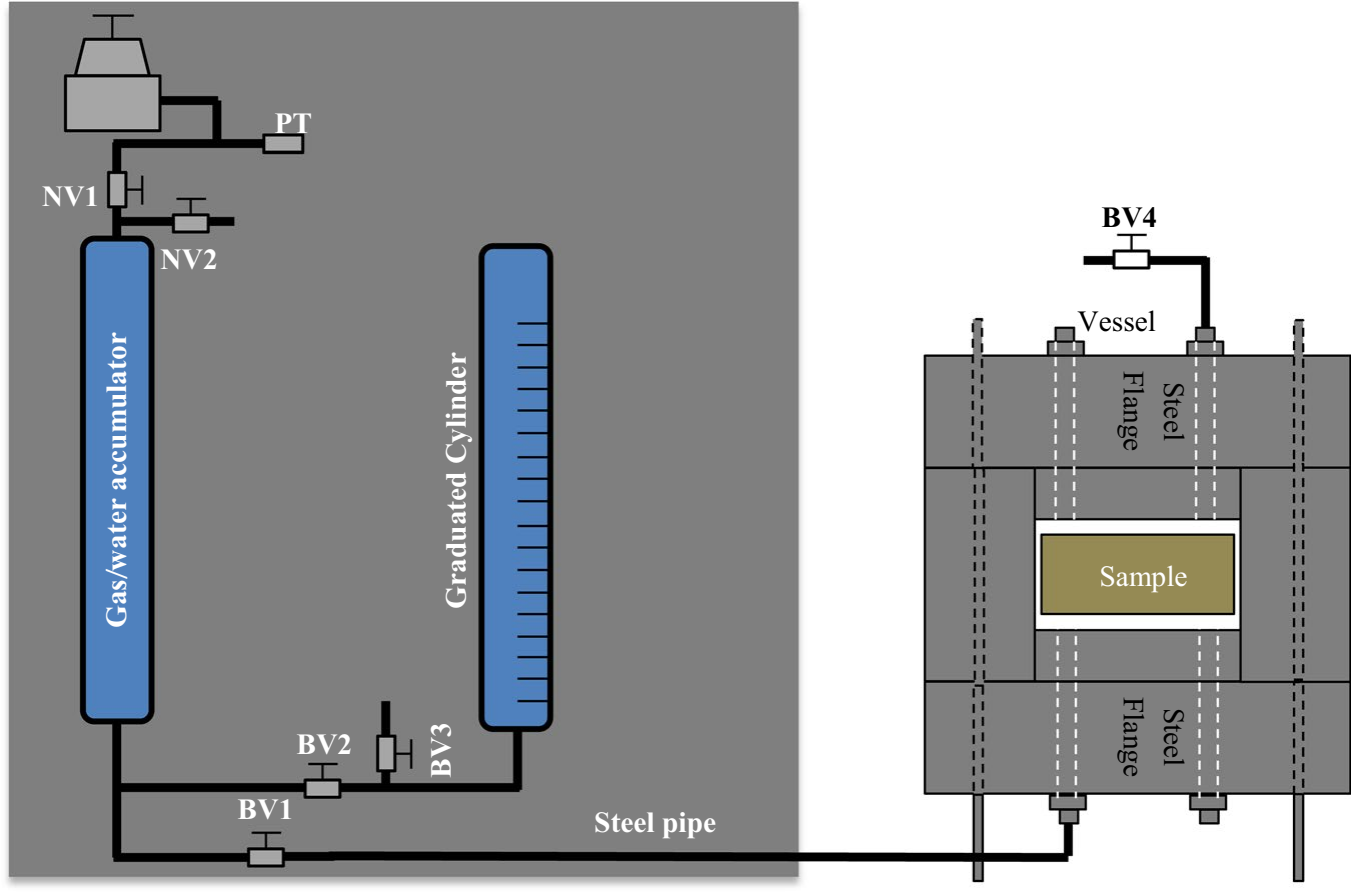
The gas/water intrusion schematic design.

The apparatus consisted of a regulator, two SWAGELOK needle valves, NV1, and NV2, four SWAGELOK ball valves, BV1, BV2, BV3, and BV4, a 300 mL gas/water accumulator, a graduated cylinder, and a steel vessel (*see* Fig. 2). The components were assembled on an aluminium plate and were connected to each other through 1/16 in. OD steel pipes. A SWAGELOK 316L Stainless Steel Double Ended DOT-Compliant Sample Cylinder, 1/4 in. FNPT, 300 cm^3^, 1800 psig was used as the gas/water accumulator. The pressure within the accumulator was recorded throughout a microgage P-102 pressure transducer connected to a data acquisition system. The pressure transducer was rated for 0 to 14 MPa with the accuracy of ±0.4 kPa. It was calibrated with a dead-load calibration apparatus. The output of the transducer was connected to a μMAC-5000 analog-to-digital converter. The converter was connected to a personal computer running LabTech Notebook version 7.2.1 W data-acquisition software. The data acquisition system displayed the pressure numerically, and graphically as a function of time. The pressures and elapsed time were recorded every second.

### Calibration test

A calibration test was performed to understand the behaviour of argon gas within the accumulator during the expansion process. The accumulator was filled partially with 200 ml of distilled de-aired water at ambient pressure and temperature. Then, argon gas was injected into the accumulator to increase the pressure to 200 kPa. At this step, the system of gas/water was in thermodynamic equilibrium. Next, the ball valve, BV1 was opened to collect a specific amount of water from the accumulator. This led the argon gas to expand within the accumulator. Then, the valve BV1 was closed, and the amount of water collected was measured using a graduated cylinder. The accumulator pressure was recorded by the data logger, which was connected to the pressure transducer, PT, to get one data point of pressure-volume. Opening and closing the valve BV1 was repeated nine times to get nine data point of pressure-volume. Finally, the calibration results depicted a P-V diagram, showing that the gas volume through time, *V*(*t*) [ml] can be related to the pressure through time, *P*(*t*) [kPa] by a polytropic process.

1 $$V(t)=\sqrt[n]{K/P(t)}$$

where *n* is the polytropic power index [−] and *K* is the polytropic constant. Their values were obtained by the best curve fitting method.

### The GEIWIP measurement technique

The gas/water intrusion set up (Fig. 2) was designed to implement the GEIWIP measurement. The purpose of the test was to determine porosity of concrete samples. This test consists of a pressurized system where the sample is wetted using de-aired water and pressure is created using argon gas.

**Figure 3:**
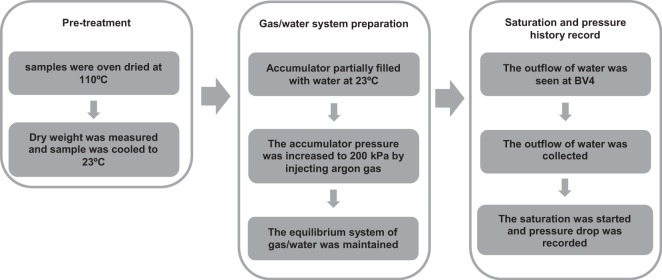
Triple steps of the GEIWIP experiment.

The optimum values of test parameters including sample size, pre-treatment time, volume of water, saturation time, temperature, and initial pressure are summarized in the triple steps flowchart in Fig. 3.

The samples were oven dried at 110 °C for 24 h to minimize the pore moisture. It was assumed that heating the concrete samples up to this temperature induces shrinkage cracks. However, this did not affect the test results, as all the samples were heated up to 110 °C a few times prior to the main tests and the potential changes of the concrete structure had already taken place. Before placing the samples in the vessel, the dry weight, *W*_*d*_, was measured and the samples were cooled to room temperature (23 °C) while wrapped with a plastic film to isolate ambient moisture. Then, the plastic film was removed and the sample was placed in the vessel. There was a small gap between the sample and the vessel inner walls to allow water to flow around the sample.

Similar to the calibration test, the accumulator was partially filled with 200 ml of distilled de-aired water at room temperature and under ambient pressure. This was done by using the U-shape system (*see* Fig. 2) connected to the accumulator, including the graduated cylinder, the ball valves BV2, BV3. Once the accumulator was partially filled, BV2 was closed. The remaining 100 ml (unfilled volume) of the accumulator initially accommodated air while argon gas was replaced with air later in the test. The accumulator and the vessel were connected through BV1 (*see* Fig. 2). With BV1 being closed, the sample was dry within the vessel. Then, argon gas was injected into the accumulator to reach the initial pressure of 200 kPa. This pressure was selected based on the complementary tests that were done with different initial pressures; higher initial pressures led to overestimation of porosity due to elastic deformation of pores within the concrete solid structure and lower initial pressures led to longer saturation time. Because the initial conditions of the tests were set to be the same as the calibration test, the same behaviour of gas (*i*.*e*. the polytropic function of calibration test) was expected during the test. The initial conditions of the calibration test were set as the initial conditions of the sample tests to simulate the behaviour of argon gas during calibration test.

Transducers were used to measure the pressure in the accumulator, which was used to determine sample saturation. Transducers were connected to the data logger, started to record the pressure of the accumulator. After the needle valve, NV1, was closed, the system of gas/water was balanced at equilibrium condition under 200 kPa in the accumulator. Next, valves BV4 and BV1 were opened, so that the water could readily flow into the vessel, allowing the argon gas to expand within the accumulator. The high pressure of water surrounding sample caused water to enter the low pressure zones of the sample with dry pores. This began the sample saturation process. The outflow of water from BV4 was collected and the volume was measured by a graduated cylinder for later calculations. Once the outflow of water with no air bubbles observed at BV4, the vessel was assumed to be filled and BV4 was closed. The saturation process of water intrusion into the sample was carried out until no more pressure drop was observed by the pressure transducer, indicating sample saturation. At the end, the vessel was dismantled and the saturated sample weight in air, *W*_*s*_ was measured.

The whole amount of water that flowed to the vessel at time *t*, *V*(*t*) was equal to the change of volume of argon during the intrusion. According to the calibrated polytropic relation between the pressure and volume, the amount of water could be calculated versus time. Finally, the amount of water that was intruded into the concrete sample, *V*_*int*_(*t*) was calculated as follows:

2 $${V}_{int}(t)=V(t)-{V}_{ann}-{V}_{out}-100$$

Here, *V*_*ann*_ [ml] is the annular volume between the sample and the vessel chamber. *V*_*out*_ [ml] is the collected outflow volume. Considering that the saturation occurred at time *T*, *V*_*int*_(*T*) [ml] was used to calculate the porosity. Knowing the bulk volume of any sample, *V*_*bulk*_ [ml], the porosity by this method, ${\varnothing }_{GEIWIP}$ [%] was determined by Eq. (3).

3 $${\varnothing }_{GEIWIP}=\frac{{V}_{int}(T)}{{V}_{bulk}}\times 100$$

The porosity measured by GEIWIP represents the pores that were filled with water. This is the total water intruded into the dried concrete sample, filling the voids, the pores within the cement paste, and the pores within the aggregates (*see* Fig. 1). The triple steps of pre-treatment, gas/water system preparation, and saturation were repeated three times (termed Test-1, Test-2, and Test-3) to get three pressure-time histories for each sample tested and evaluate variations of results. For each concrete sample with a specific porosity, the saturation time T varies with the saturation pressure *P*_*s*_ and the viscosity μ of the intrusive fluid (*i*.*e*. distilled de-aired water) at the intrusion temperature (*i*.*e*. 23 °C). It was noted that any changes in room temperature would change the viscosity and in turn would alter the saturation pressure and time for each sample. In order to get an independent parameter of either pressure or temperature, the non-dimensional saturation time T^*^ is defined in Eq. (4). This parameter was used to compare the saturation time of any sample regardless of saturation pressure or temperature.

4 $${{\rm{T}}}^{\ast }={P}_{s}\cdot \frac{{\rm{T}}}{{\rm{\mu }}}$$

### The saturation test

**Figure 4:**
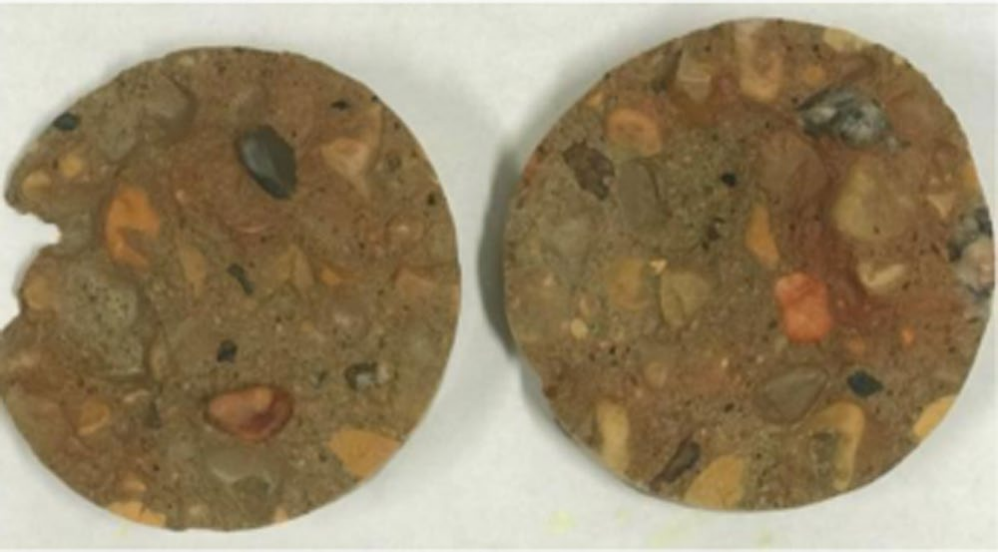
Concrete sample cut after saturating process with dyed DDW through GEIWIP test.

In this experiment the sample is not vacuumed for two reasons. First, the intruding fluid is a wetting fluid (i.e. DDW), so that the capillary forces may intrude water into the air-filled interconnected pores and drive the air to the outer surface or to the dead-end pores. Second, the applied pressure during the saturating process may decrease the volume of trapped air in dead-end pores and that increases the possibility of water intrusion into the dead-end pores. Yet, there will be a portion of pores occupied with air, which comparing to the large bulk volume of the samples (50 mm in diameter of cylindrical samples), this amount is assumed negligible. To ensure that this assumption is correct, a GEIWIP test was performed using a dyed DDW with the Rhodamine. Once the saturating process was completed the vessel was dismantled and the sample was cut. Figure 4 shows that the dyed DDW could get intruded through the sample’s inner pore structure indicating the full saturation of the sample. Additionally, a comparison of incremental pores volume between the GEIWIP experiment and NMR result at 100% saturation is shown in the Supplementary Fig. S1 for all samples. The comparison shows a good estimation of sample’s saturation in the GEIWIP experiment.

### Other porosimetry measurements

The GEIWIP results were compared with other porosimetry methods, including modified gas expansion, MIP, and NMR techniques. In this study, the same apparatus was utilized for the modified gas expansion method. In the common gas expansion method^24^, the process of expansion of gas was isothermal and grain volume was calculated from Boyle’s law^24^. However, in the modified gas expansion test, the expansion process was not necessarily isothermal. Similar to the GEIWIP method, a polytropic process was the governing process during the gas expansion. Therefore, prior to the main test, a calibration test was required to determine the polytropic index and polytropic constant. After determination of the polytropic equation, the main test was carried out. The accumulator (Fig. 2) was filled with argon gas to the initial pressure *P*_*i*_, while it was connected to the vessel, encompassing the sample, through the closed valve BV1. The vessel was at ambient pressure. Three tests were carried out for each sample at different initial pressures of the accumulator ranging from 83–92 kPa. Once the BV1 was opened, the equilibrium pressure *P*_*e*_ was measured by a pressure transducer PT. Then, knowing the polytropic index *n*, the grain volume *V*_*grain*_, was calculated from Eq. (5).

5 $${V}_{grain}=(1-\sqrt[n]{\frac{{P}_{i}}{{P}_{e}}}\,){V}_{i}+{V}_{vessel}$$

where *V*_*i*_ and *V*_*vessel*_ are initial and vessel volume, respectively. As a result, the porosity from the modified gas expansion method ${\varnothing }_{GE}$ of the sample was calculated as follows:

6 $${\varnothing }_{GE}=(1-\frac{{V}_{grain}}{{V}_{bulk}})\times 100$$

In addition, the GEIWIP results were compared with MIP results. MIP tests were carried out on a similar concrete samples. However, the size of each sample had a maximum 5 mm diameter and only the mortar part of the concrete (excluding coarse aggregates) was placed in the MIP apparatus due to its spatial limitation. The details of the MIP tests on these concrete samples can be found in^23^.

Additionally, NMR was carried out on exactly similar concrete samples. In this test, the relaxation behaviour of magnetically excited water in the concrete sample was investigated. In the presence of a magnetic field gradient, water was allowed to diffuse into the concrete sample by its weight and under ambient pressure. The transverse dephasing pulse, known as T_2_ relaxation time (ms), was recorded while the sample was saturating. The T_2_ relaxation time was measured with an interecho spacing of 0.1 ms and a minimum signal to noise ratio (SNR) of 100:1, using an Oxford Maran DRX-HF instrument at 30 °C and 2 MHz frequency coupled with GITSystems software. T_2_ distribution curves obtained by the software at 100% saturation were used to calculate the pore volume and porosity.

Furthermore, once the GEIWIP test was completed, the measured weight of the samples at dry and saturated conditions provided another porosity measurement that was calculated by Eq. (7). Weights were measured in a balanced set-up (Mettler Toledo XSTM, accuracy of 0.01 mg).

7 $${\varnothing }_{w}=\frac{({W}_{s}-{W}_{d})/{\rho }_{{H}_{2}O}}{{V}_{bulk}}$$

The density of distilled de-aired water, *ρ*_*H2o*_, at room temperature of 23 °C was measured and porosity ${\varnothing }_{w}$ was calculated for all samples at each test.

## Results and Discussion

### Calibration test results

**Figure 5:**
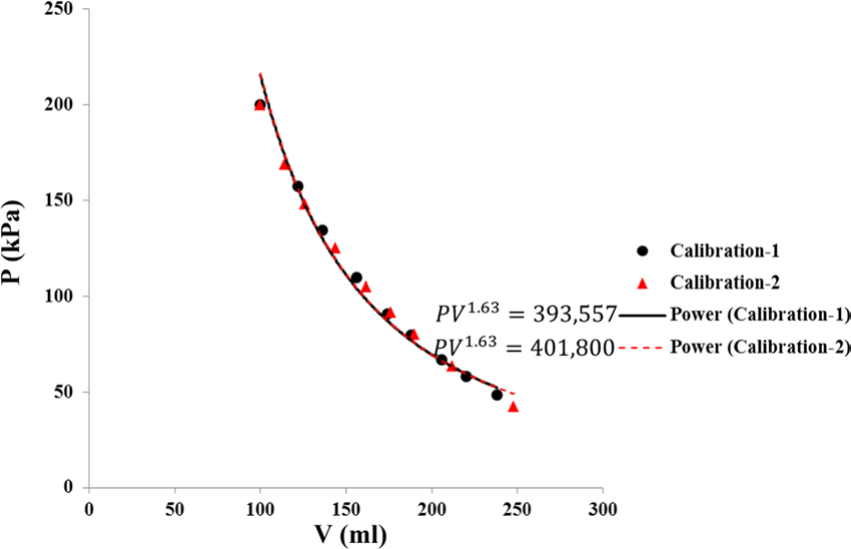
Data points from the calibration tests starting at initial state (200 kPa, 100 ml) and their corresponding power law function curve fittings 1 and 2.

To calibrate the polytropic process of argon gas at the initial state (*i*.*e*. 200 kPa pressure, temperature of 23 °C, and water volume of 100 ml, nine data points were recorded as water was released from the system and pressure decreased. The resulting curves are presented in Fig. 5. The data points were obtained two times (*i*.*e*. before and after the test). A power curve was fit to each set of data points, depicted with solid black and dashed red lines as Power (calibration-1 and -2) in the P-V diagram given in Fig. 5.

The calibration tests showed that argon gas within the accumulator was going through a polytropic process, termed the “GEIWIP pressure-volume relationship”. Two curve fittings resulted in a polytropic index of 1.63, and two different constants presented in Fig. 4. The absolute average error for the power law curve of Calibration-1 was −2.0 % and for the power law curve of Calibration-2 was 7.5%, hence, the function for Calibration-1 was considered as the GEIWIP pressure-volume relationship.

The constant with less cumulative error percentage was selected to form the GEIWIP relationship as shown in Eq. (8).

8 $$V(t)=\sqrt[1.63]{393557/P(t)}$$

### The GEIWIP test results

**Figure 6:**
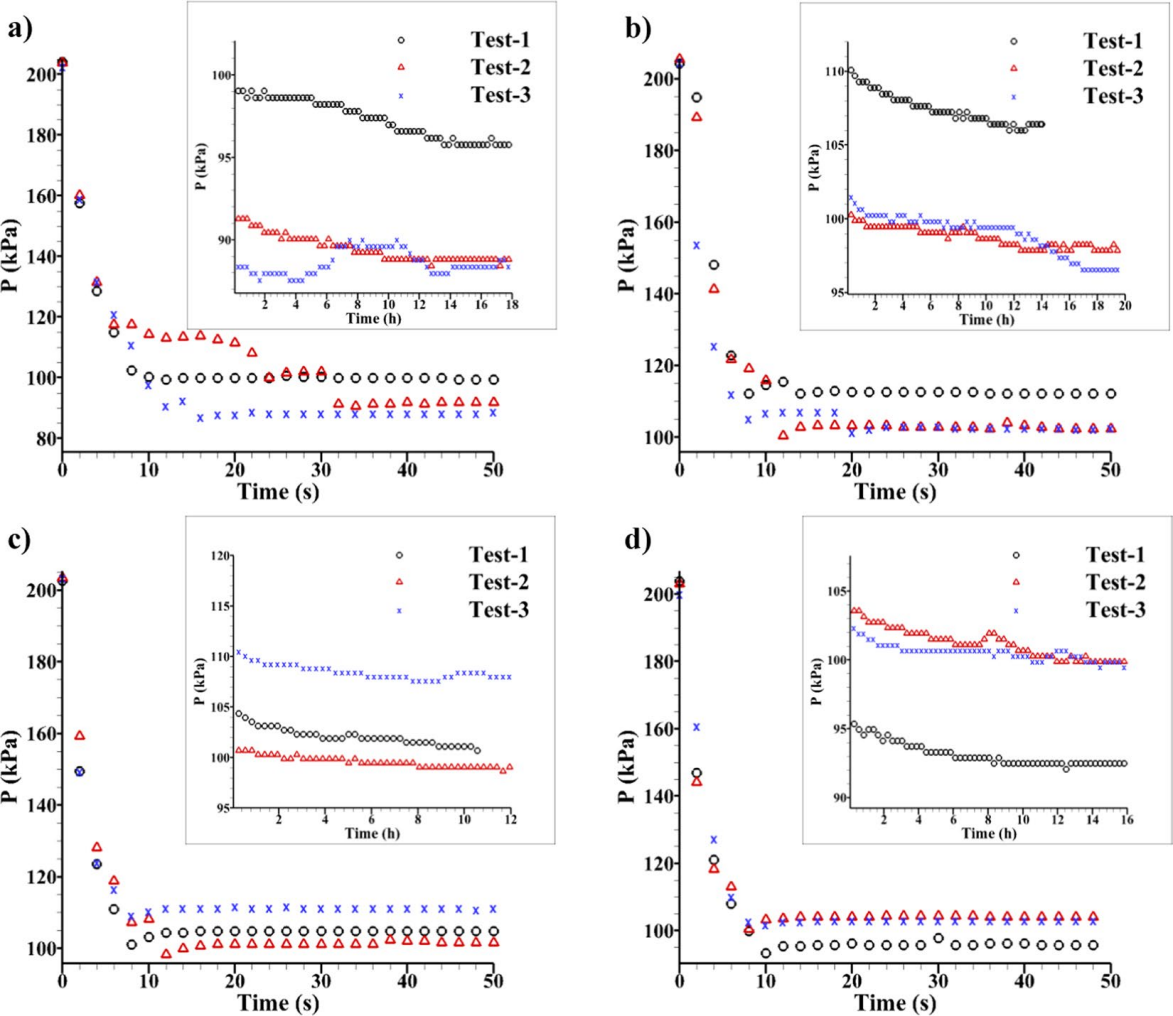
GEIWIP pressure-time plot for samples: (**a**) GUF20, (**b**) GUF30, (**c**) PLCF30, and (**d**) PLCF30S. Inset plots showing the saturation status after 18, 20, 12, and 16 hours, respectively.

During the saturation process, pressure-time data points were recorded until no pressure reduction was noted, which was considered as the saturated condition for each sample. Figure 6a–d show the pressure-time plots including the first 50 seconds, and from 50 seconds to 20 hours (inset plots) for sample GUF20, GUF30, PLCF30 and PLCF30S, respectively. The GEIWIP test was repeated three times on the same samples and the corresponding pressure-time data points are depicted with black circles for Test-1, red triangles for Test-2, and blue crosses for Test-3, in Fig. 6. It shows that first the pressure of argon gas within the accumulator dropped from the initial state of 200 kPa due to the flow of distilled de-aired water to the vessel. This pressure drop involved with the flow of water to the annular volume and filling this volume, its intrusion to the pores of the concrete sample, and the outflow volume. Different pressure drops were seen for Tests 1 to 3, as a result of different outflow volumes. For example, in Fig. 5a, the outflow of water taken for Test-1, was 30 ml, while for Test-2 and Test-3 the values were 39 and 42 ml, respectively. Therefore, Test-2 and Test-3 show more pressure drop than Test-1 for the first 50 seconds of the experiment (*see* Fig. 6).

The volume of water outflow for each test was used to calculate the intruded volume in the sample, which is the pore volume filled with water. The experiment continued until the time that the pressure remained constant, noted as saturated pressure ${P}_{s}$, and saturated time ${T}_{s}$ (Fig. 6). The saturated pressure-time of Test-1 was 95.8 kPa after 15 h. For Test-2 and Test-3, the saturated pressure-times were 88.8 kPa after 14 h and 88.4 kPa after 14 h, respectively (Fig. 6a). Similarly, in Fig. 6b, the outflow of water taken at Test-1 was 20 ml, while at Test-2 and Test-3, it was 28 ml, equally. Therefore, Test-2 and Test-3 showed more pressure drop than Test-1 within the first 50 seconds of the experiment. The experiment continued until the saturated state. The saturated pressure-time of Test-1 was 106.4 kPa after 14 h. For Test-2 and Test-3, the saturated pressure-times were 97.8 kPa after 19 h and 96.5 kPa after 19 h, respectively.

**Figure 7:**
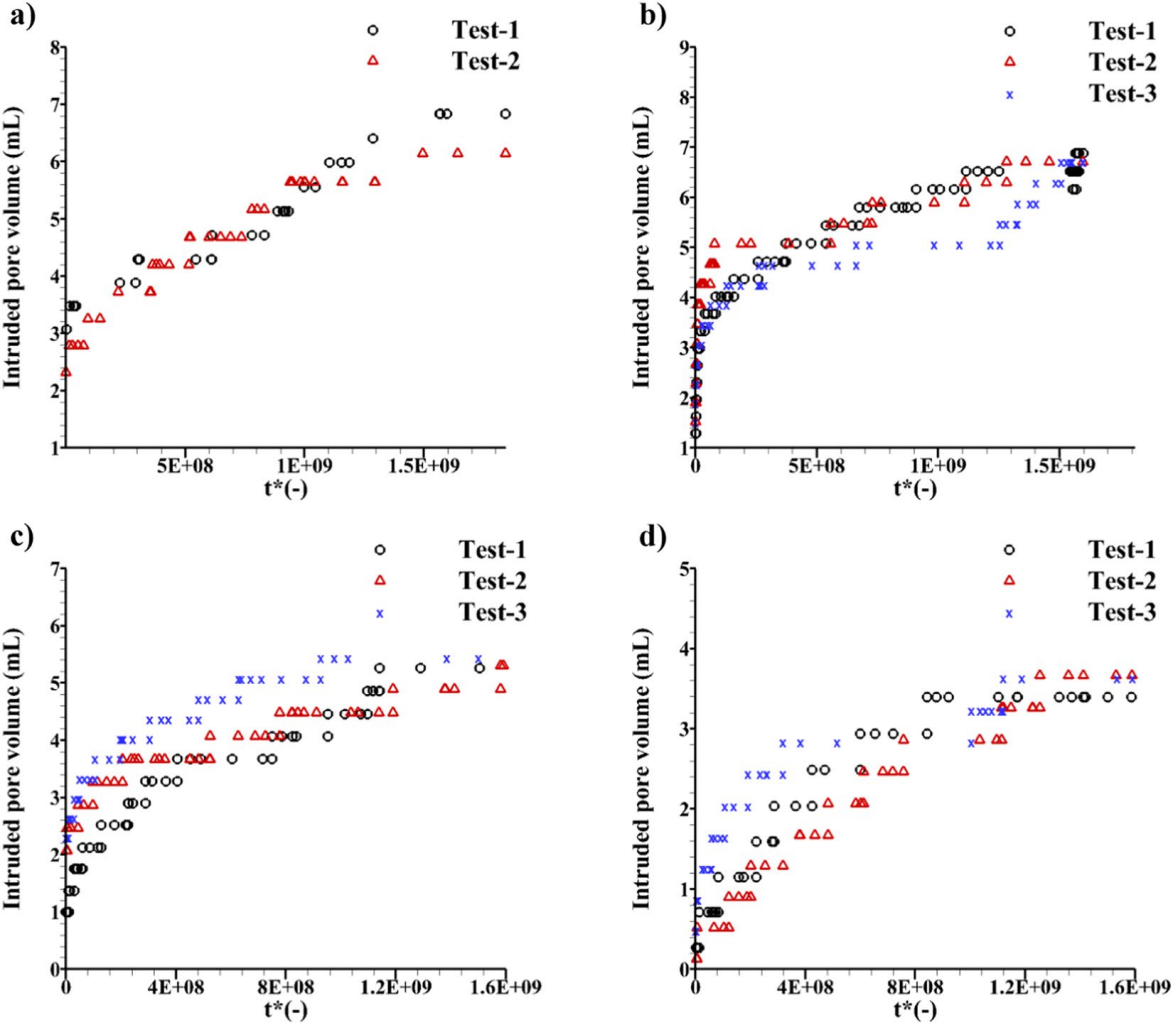
Intruded pore volume data points of the GEIWIP test for samples: (**a**) GUF20, (**b**) GUF30, (**c**) PLCF30, and (**d**) PLCF30S.

Finally, pressure-time data points were substituted into Eq. (8). Then, Eq. (2) was used to obtain intruded volume *V*_*int*_. The annular volume, *V*_*ann*_ between the sample and the vessel for sample GUF20, GUF30, PLCF30, and PLCF30S were 28 ml, 28 ml, 29 ml, and 33 ml, respectively. The intruded volume was plotted versus non-dimensional time t^*^ in Fig. 7. It is indicated that for each sample, non-dimensional saturation time t^*^ was equal in Test-1 to 3. For example, in Fig. 7a, the saturation was obtained at ${t}^{\ast }=1.49\,\times {10}^{9}$ in Test-1 and 2 of sample GUF20. For sample GUF30 (Fig. 7b), saturation was seen at ${t}^{\ast }=1.50\,\times {10}^{9}$. For samples PLCF30 (Fig. 7c) and PLCF30S (Fig. 7d), saturation was obtained at ${t}^{\ast }=1.57\times {10}^{9}$ and ${t}^{\ast }=1.60\,\times {10}^{9}$, respectively. As t^*^ was shown to be independent of test conditions such as initial pressure, or outflow volume, it can be inferred that t^*^ is an intrinsic property of each sample, showing how fast a sample is saturated. The higher the pore volume, the larger the non-dimensional saturation time determined in the GEIWIP test. The pore volume of each sample was considered as the maximum intruded pore volume (Fig. 7).

### Porosity measurement results

The interface between gas/water within the accumulator was circular and stable in shape, during the intrusion in the GEIWIP test. The pressure of the gas on the gas/water interface provided enough force for water to intrude into the pores of the sample. During the intrusion of water (*i*.*e*. saturating process), the movement of the water into the sample resulted in a decrease of water’s volume in the accumulator, leading to argon gas expansion. The expansion of argon was resulted the pressure reduction in the accumulator. The magnitude of the pressure drop was related to the volume of water intruded into the pores, according to the calibration test. Therefore, a time-pressure data was recorded until the time that no more decrease in pressure was investigated. The time and pressure values at saturated condition are denoted as saturation pressure and saturation time, respectively. A non-dimensional saturation time T^*^ was defined based on the saturation pressure and time to evaluate how fast a sample was saturated. The bulk volume was obtained from direct mathematical calculation of a cylinder volume, as the samples had a cylindrical shape. The outflow volume of water, *V*_*out*_ was collected to remove the air bubbles from the vessel. Comparison between Test-1, Test-2, and Test-3 showed consistent porosity values ${\varnothing }_{GEIWIP}$ (Table 2). As an alternative test, the porosity based on Eq. (7) was calculated and denoted as ${\varnothing }_{w}$ and compared with those of the GEIWIP method. Density of distilled de-aired water *ρ*_*H*2*O*_ at room temperature before and after the test was measured to be 0.995 and 0.992 g/cm^3^, respectively. The change in density was observed due to the dissolution of gas into the water, thus, water was expanded 0.3% of its initial state, at the end of the experiment. The mean value of density 0.9935 g/cm^3^ was substituted in Eq. (7) to calculate the porosity. Comparison between Test-1, Test-2, and Test-3 shows consistent porosity values.

**Table 2:** The GEIWIP test parameters and porosity measurement results.

Sample ID.	*V*_*bulk*_ (mL)	Test #	*V*_*out*_ (mL)	*V*_*pore*_ (mL)	${{\boldsymbol{\varnothing }}}_{{\boldsymbol{GEIWIP}}}$ (%)	${\bar{{\boldsymbol{\varnothing }}}}_{{\boldsymbol{GEIWIP}}}$ (%)	S	*W*_*d*_ (g)	*W*_*s*_ (g)	${{\boldsymbol{\varnothing }}}_{{\boldsymbol{w}}}$	${\bar{{\boldsymbol{\varnothing }}}}_{{\boldsymbol{w}}}$
GUF20	38	1	30	6.84	18.0	18.4	0.56	77.43	84.37	18.4	18.3
		2	39	7.14	18.8			77.43	84.34	18.3	
		3	42	—	—			77.36	84.26	18.3	
GUF30	38	1	20	6.88	18.1	17.8	0.23	78.57	84.02	14.4	14.4
		2	28	6.71	17.7			78.57	84.00	14.4	
		3	28	6.71	17.7			78.64	84.04	14.3	
PLCF30	37	1	26	5.26	14.2	14.4	0.26	81.25	85.81	12.4	12.4
		2	28	5.30	14.3			81.25	85.84	12.5	
		3	19	5.44	14.7			81.36	85.89	12.3	
PLCF30S	33	1	32	3.39	10.3	10.8	0.44	72.60	76.09	10.6	10.4
		2	24	3.66	11.1			72.66	76.03	10.3	
		3	24	3.63	11.0			72.65	76.03	10.3	

In order to check the robustness of the GEIWIP test, the standard deviations (i.e. uncertainty), S of the results was obtained by doing multiple tests (three tests in this study) on the same samples. The standard deviations of porosities for each sample were varied between 0.23 to 0.56. The mean value of porosities obtained from the three tests for each sample were denoted as ${\bar{\varnothing }}_{GEIWIP}$. The comparison between mean porosity ${\bar{\varnothing }}_{w}$ and ${\bar{\varnothing }}_{{\rm{GEIWIP}}}$ showed a good agreement with a maximum difference of 0.1%, 3.4%, 2%, and 0.4% for sample GUF20, GUF30, PLCF30, and PLCF30S, respectively. The difference observed between ${\bar{\varnothing }}_{w}$ and ${\bar{\varnothing }}_{{\rm{GEIWIP}}}$ for samples GUF20 and PLCF30S is less than the standard deviation, S, of the corresponding porosity measurement in GEIWIP test (see Table 2, S values). Thus, the effect of experimental error is negligible for those samples.

The accuracy of the experiments was evaluated through the comparison with modified GE, NMR, and MIP tests. The modified GE was performed using the same apparatus without water, using argon gas as the working fluid. The resultant porosities were in a good agreement with NMR porosities. The results indicated that the GEIWIP test was a useful apparatus to provide porosity data with and acceptable range of accuracy. The costs and duration of each run are significantly lower, compared to NMR and MIP tests.

### Comparison of the GEIWIP results with modified GE results

The GEIWIP results were compared with the results obtained from modified gas expansion (GE) technique. The measurements were done on exactly the same concrete samples. A graduated cylinder was used to measure the vessel volume to be 66 mL.

It was noticed that the pressure ratio, *P*_*i*_/*P*_*e*_ in all tests 1–6 is constant (Table 3). According to Eq. (6), when the vessel is empty, the grain volume is zero so that the pressure ratio must be proportional to the volume ratio *V*_*vessel*_/*V*_*i*_. As the volume ratio was the same (*i*.*e*. 0.22) in all tests 1–6, the resultant pressure ratio was constant. Each test was completed in 5 minutes. Finally, a polytropic index of 1 was obtained from solving Eq. (6). The polytropic index of unity stands for an isothermal process. Therefore, Boyle’s law was valid for the modified gas expansion test.

**Table 3:** Calibration test prior to modified GE test.

Test	*P*_*i*_ (KPa)	*P*_*e*_ (KPa)	*P*_*i*_/*P*_*e*_	*n*
1	51.18	41.77	1.22	1
2	101.93	83.51	1.22	
3	150.64	123.62	1.22	
4	201.38	164.96	1.22	
5	300.84	246.40	1.22	
6	400.00	327.44	1.22	

Once the behaviour of gas was recognized to be isothermal, the modified GE experiment was started. The operating pressure was in the range of 83 to 92 kPa in Test-1, Test-2, and Test-3. This range of pressure was selected to have a similar saturation condition as the GEIWIP test. The mean value of three porosities was calculated and reported as ${\bar{\varnothing }}_{GE}$ (Table 4). The mean porosity of each sample was compared with the GEIWIP mean porosity ${\bar{\varnothing }}_{GEIWIP}$. The comparison shows that ${\bar{\varnothing }}_{GE}$ was in good agreement with ${\bar{\varnothing }}_{GEIWIP}$. This was because of the water in the GEIWIP test, which is a wetting fluid, tending to penetrate from larger pores to smaller pores due to capillary forces. In the GEIWIP test, the capillary forces within the pores are toward the flow of water intrusion. Therefore, in smaller pores, where capillary forces are larger, the water may continue to flow and fill the pores. On the other hand, in the modified GE test, argon gas is a non-wetting fluid and the capillary forces are against the flow and oppose the intrusion of gas. In pore throats, capillary forces are large enough to prevent the gas from filling the pores. However, argon gas had lower viscosity compared to water in GEIWIP test. The low viscosity feature of gas overcame the capillary forces and penetrated to pore throats and provided the same porosity as GEIWIP porosity.

**Table 4:** Results of modified GE and their comparison with GEIWIP porosity.

Sample ID	Test #	*P*_*i*_ (KPa)	*P*_*e*_ (KPa)	*P*_*i*_/*P*_*e*_	*V*_*grain*_ (mL)	Porosity, ${{\boldsymbol{\varnothing }}}_{{\boldsymbol{GE}}}$	S	${\bar{{\boldsymbol{\varnothing }}}}_{{\boldsymbol{GE}}}$	${\bar{{\boldsymbol{\varnothing }}}}_{{\boldsymbol{GEIWIP}}}$
GUF20	1	87.16	78.16	1.115	31.45	17.2	1.07	18.1	18.4
	2	89.20	79.80	1.118	30.66	19.3			
	3	90.84	81.43	1.116	31.20	17.9			
GUF30	1	83.47	74.88	1.115	31.50	17.1	0.80	17.9	17.8
	2	86.34	77.34	1.116	31.20	17.9			
	3	90.02	80.61	1.117	30.90	18.7			
PLCF30	1	87.16	78.15	1.115	31.50	14.9	0.52	14.6	14.4
	2	90.88	81.43	1.115	31.50	14.9			
	3	91.66	82.24	1.114	31.80	14.0			
PLCF30S	1	88.38	78.96	1.119	30.30	8.1	2.75	10.9	10.8
	2	86.07	76.93	1.122	29.40	10.9			
	3	92.07	81.84	1.125	28.50	13.6			

### Comparison of the GEIWIP results with NMR and MIP results

**Figure 8:**
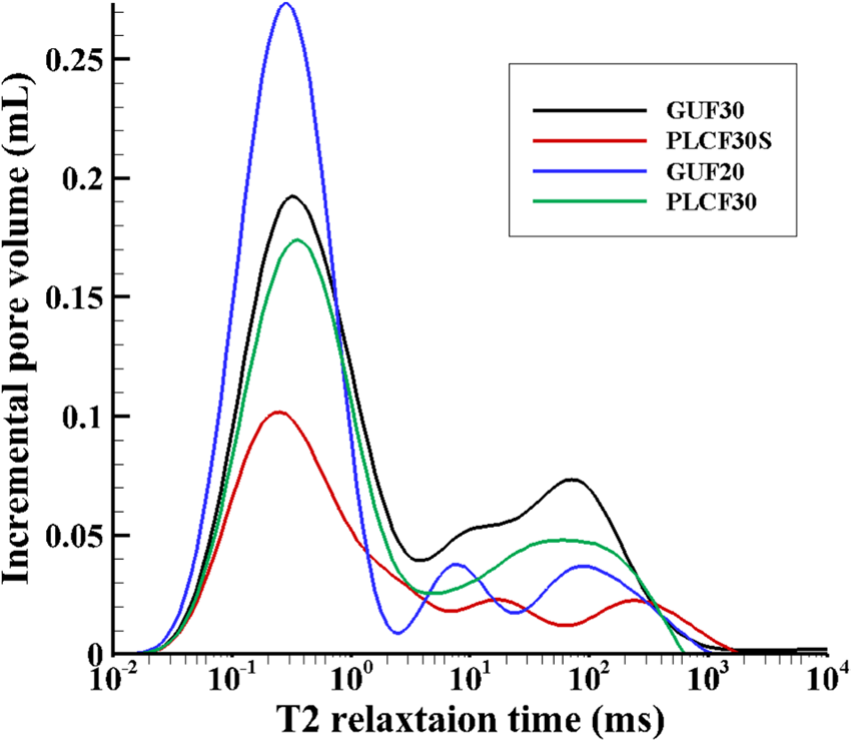
T_2_ distribution curves at 100% saturation in NMR for samples GUF20, GUF30, PLCF30, and PLCF30S.

In the NMR tests, the ${{\rm{T}}}_{2}$ relaxation time was measured. The ${{\rm{T}}}_{2}$ distribution curve (Fig. 8) was used to calculate the summation of all incremental volumes, considered as the cumulative pore volume. Subsequently, the mean GEIWIP porosity of each sample was compared with the porosity obtained from NMR, ${\varnothing }_{NMR}$ and presented in Table 5. A good agreement was seen between the NMR and GEIWIP porosity values. This confirms that the samples during the GEIWIP test were fully saturated (*see* Supplementary Fig. S1). The differences of ${\bar{\varnothing }}_{GEIWIP}$ and ${\varnothing }_{NMR}$ ranged between 1 to 8%.

**Table 5:** Results of NMR test and its comparison with GEIWIP porosity.

Sample ID	NMR cumulative pore volume (mL)	${{\boldsymbol{\varnothing }}}_{{\boldsymbol{NMR}}}$	GEIWIP Mean pore volume (mL)	${\bar{{\boldsymbol{\varnothing }}}}_{{\boldsymbol{GEIWIP}}}$
GUF20	6.48	17.0	6.99	18.4
GUF30	6.68	17.6	6.77	17.8
PLCF30	5.43	14.7	5.33	14.4
PLCF30S	3.31	10.0	3.56	10.8

Finally, MIP tests on the concrete samples with the same type of binders were conducted to determine the apparent porosity. The results were reported in^23^ and are summarized in Table 6. The MIP results show 9–33% lower porosities comparing to NMR and GEIWIP results. MIP porosities were reported as the apparent porosity, representing only the pore volume, which were inter-connected to each other and to the surface of the concrete cores. However, in the NMR and the GEIWIP tests, water, a wetting fluid, intruded beyond the pore throat. This led to higher porosities by the NMR and the GEIWIP tests compared to the MIP porosity. In addition, the higher GEIWIP porosities can be explained by capillary forces. In the GEIWIP test, the capillary forces within the pores are toward the flow of water intrusion. Therefore, in smaller pores, where capillary forces are larger, the water may continue to flow and fill the pores. In the MIP test, the capillary forces are against the flow and oppose the intrusion of mercury. In smaller pores, capillary forces are large enough to prevent the mercury from filling the pores. Hence, the GEIWIP porosity represents the volume of all pores and the concrete samples were saturated within 20 hours, while the MIP porosity includes only inter-connected pores volume.

**Table 6:** Results of MIP test and its comparison with modified GE and GEIWIP porosity.

Sample ID	${{\boldsymbol{\varnothing }}}_{{\boldsymbol{MIP}}}$ (Ghazy and Bassuoni, 2018)	${{\boldsymbol{\varnothing }}}_{{\boldsymbol{NMR}}}$	${\bar{{\boldsymbol{\varnothing }}}}_{{\boldsymbol{GE}}}$	${\bar{{\boldsymbol{\varnothing }}}}_{{\boldsymbol{GEIWIP}}}$
GUF20	15.6	17.0	18.1	18.4
GUF30	16.2	17.6	17.9	17.8
PLCF30	13.1	14.7	14.6	14.4
PLCF30S	7.2	10.0	10.9	10.8

## Conclusions

This study utilized an alternative Gas Expansion Induced Water Intrusion Porosimetry (GEIWIP) method to measure the porosity of consolidated porous samples. Four concrete cores with different binder types were used as test samples. The gas/water intrusion apparatus provided a simple procedure based on the thermodynamic behaviour of the gas (argon), stored in the accumulator at a certain pressure (200 kPa), to study the pore volume of the samples. The robustness of the GEIWIP test was confirmed according to the standard deviations of each sample. The comparison of the results to other porosimetry methods showed the reliability of the test. The advantages of the GEIWIP test over MIP or GE tests is the use of water instead of mercury or gas as intruding fluid that is hazard free and gets access to all interconnected pores. It provided access to the finer pores and to the large pores beyond the narrow throats, thus, the measured porosities were larger than those obtained from MIP tests. In spite of MIP test, the GEIWIP test is non-destructive, low cost and safe to implement. The GEIWIP test showed the similar porosity values as the NMR test at a lower cost and easy and safe implementation steps. In the GEIWIP test, samples can be selected large enough to be a good representative of the whole geometrical features. The GEIWIP’s time-pressure data provides the pressure reduction history that shows the range of the pore sizes getting filled during the saturating process. The initial pressure to inject water into the sample can be increased, to minimize the saturation time.

## Supplementary information

Supplementary Information
