# Supplementary material for: Porosity Measurement of Low Permeable Materials Using Gas Expansion Induced Water Intrusion Porosimetry (GEIWIP)
Source: Sci Rep. 2019 Nov 26;9:17554. doi: 10.1038/s41598-019-53441-6 (PMC6879464; doi:10.1038/s41598-019-53441-6)
Supplement: Supplementary file 1 — Supplementary Information [file 41598_2019_53441_MOESM1_ESM.pdf]

Porosity Measurement of Low Permeable Materials Using Gas Expansion Induced Water Intrusion Porosimetry (GEIWIP)

Miad Jarrahi*[a], Douglas W. Ruth[c], Mohamed T. Bassuoni[b], Hartmut M. Holländer[b]

[*a]*Geotechnical laboratory, Department of Civil Engineering, University of Manitoba, Winnipeg, Manitoba, Canada*
[b]*Department of Civil Engineering, University of Manitoba, Winnipeg, Manitoba, Canada*
[c]*Department of Mechanical Engineering, University of Manitoba, Winnipeg, Manitoba, Canada*
[*]***Corresponding author email: jarrahim@myumanitoba.ca***

A comparison of incremental pores volume between the GEIWIP experiment and NMR result at 100% saturation is shown in the Supplementary Fig. S1 for all samples. The underestimation of incremental pores volume in the GEIWIP test shows that those pores are missed to get saturated in comparison with NMR test. In sample GUF20, it is observed that the GEIWIP test overestimates the volume of the capillary pores and macro pores that resulted in 1.4% higher porosity value than NMR test. In sample GUF30, the GEIWIP test overestimates the volume of capillary pores but underestimates the volume of macro pores that resulted in 0.2% higher porosity value than NMR test. The same behaviour is observed for samples PLCF30 and PLCF30S. It is shown that the GEIWIP test provides the incremental pores volume of all ranges of pore sizes in concrete samples from finer pores (*i.e.* gel pores) to capillary pores and macro pores. Therefore, this comparison shows a good estimation of sample's saturation in the GEIWIP experiment.

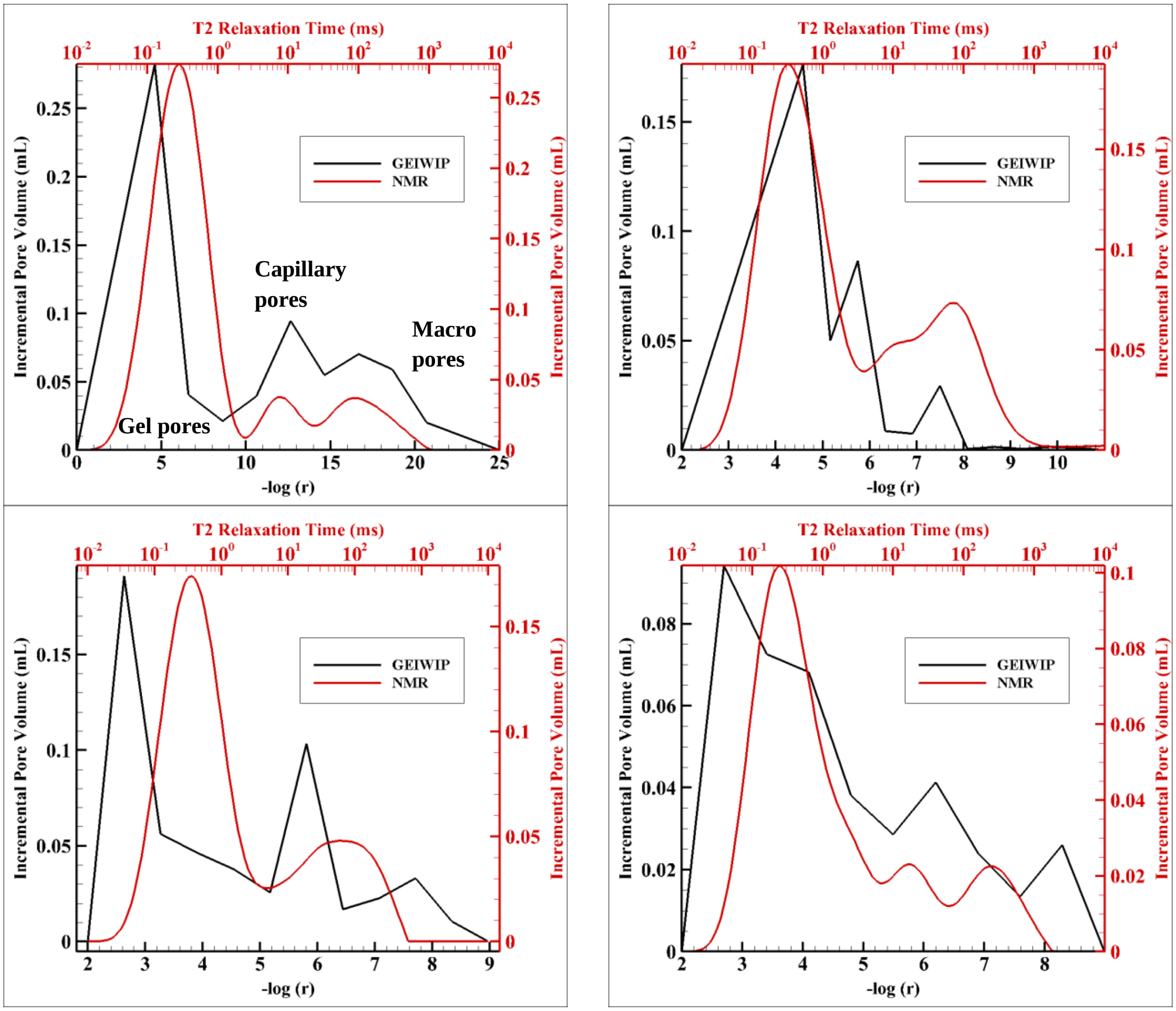

Supplementary Fig. S1 Incremental pore volume distribution in GEIWIP and T_2 distribution curve at 100% saturation in NMR for sample a) GUF20, b)GUF30, c)PLCF30, d)PLCF30S.
